# Chicago Medical Society

**Published:** 1886-07

**Authors:** 


### Society Reports.

### CHICAGO MEDICAL SOCIETY.

Officially reported for the ATLANTA MEDICAL AND SURGICAL JOURNAL.

STATED MEETING, May 17, 1886.

The President, E. J. Doering, M. D., in the chair.

Dr. Edmund Andrews exhibited a

### NEW EVACUATOR FOR LITHOLOPAXY.

Dr. Andrews said one serious defect of Bigelow's and Thompson's evacuators is the rubber bulb makes suction only for an instant instead of continuously, thus allowing fragments of stone which lie along the tube to be thrown back into the bladder when the bulb is compressed, thus irritating the bladder as well as causing it to be repeatedly expanded. To obviate these difficulties Dr. Andrews has had constructed an evacuator in which the essential features consist in a double-chamber evacuating tube, straight or curved, the upper chamber being composed of thin metal 8½ millimetres in diameter, terminating in a round tip with fenestrum for evacuating, the under chamber being semi-cylindrical, fitting under the upper chamber so as to make the whole tube oval-shaped, with a diameter of 31½ millimetres. To the under or inflow tube is attached a rubber tube three yards in length, with an inside diameter of one centimetre, to the outer end of which is fitted a metallic strainer large enough to admit without resistance all the water which will flow through the tube. A bucketful of warm carbolized water, 1½ per cent., is hung over the operating table high enough for the surface of the water to be forty-two inches above the pubis of the patient. When it is desired to wash out the bladder, the evacuating tube is introduced with the fenestrum towards the patient's head and the tip of the tube pressing towards the rectum, thus making a hollow in the bladder into which the fragments of stone fall. The strainer having previously been introduced into the warm carbolized solution and the rubber tube filled, this tube is attached to the inflow tube and the stop-cock turned. The water now enters the inflow tube and passes into the bladder through the small perforations with sufficient force to rapidly drive the fragments of stone through the fenestrum and out of the evacuator. The current is continuous and rapid; in a recent case of litholopaxy, where the calculus was hard oxalate of lime an inch in diameter, the fragments were washed out in ten seconds. When the inflow tube is closed by a fragment of stone in the fenestrum, the operator simply presses on the last four inches of this tube, which is of rubber, and this dislodges the fragment. Dr. Andrews also called attention to a device for securing to any instrument filiform bougies by means of a split screw passing over the bougie secured by a nut, both fitting into the outer end of the catheter.

Dr. W. T. Belfield opened the discussion by saying that the disadvantages of the ordinary evacuating apparatus mentioned by Dr. Andrews were well recognized, and that the apparatus exhibited seemed adapted to remove them. It possessed, however, one element of danger, namely, the possibility of undue distension of the bladder through sudden clogging of the exit tube. In cases of concentric hypertrophy, where the bladder can contain only three or four ounces, the continuation of the powerful inflow might, if the exit were obstructed, cause serious damage. Of course if the clogging is immediately discovered, a prompt use of the stop-cock would prevent injury.

As to the urethral instrument, he would wish to be sure that the filiform could not be detached beyond the stricture. This danger attaches, of course, to all methods of securing the filiform, but would seem to be especially great in the arrangement exhibited. The loss of a filiform in a tight stricture is an extremely uncomfortable accident; in several cases surgeons have even made external urethrotomy to recover it.

To Dr. Andrews' remark that in case of such an accident urethrotomy is readily avoided by inserting a small lithotrite into the bladder and seizing the filiform, Dr. Belfield replied that when there is present a stricture so tight as to require patient work to persuade a filiform to pass, a small lithotrite could hardly enter the bladder unless reinforced by a sledge-hammer. If the urethra is everywhere large enough to admit a small lithotrite, there could be no danger of losing a filiform, because there would be no occasion to use one.

Dr. Fenn wished to know if the momentum could be increased by increasing the specific gravity of the fluid, what would be the effect of a greater elevation of the reservoir?

Dr. Andrews said that the attachment of a flexible guide to the urethrotome is always a source of care lest it become detached. This was less liable to occur with his instrument than in the old way. In case it should happen the lost guide could be seized and drawn out with a lithotrite.

The question of the pressure of the fluids and the strength of the bladder is one of great interest. But little is known about it. No more force should be used on a distended bladder, certainly, than a normal one could readily endure. We get the measure of this, to some extent, in the pressure of the expulsive power of the organ, which is a safe limit to keep inside of in the absence of other data. The pressure which Bigelow's evacuator ordinarily gives may form another guide, as this is known to be harmless. The reservoir must not be placed so high as to cause dangerous distension, should the outflow tube become obstructed. In practice forty inches had given all the current needed, and this was doubtless a safe pressure to use in any and all cases.

Dr. James I. Tucker read a paper on

UNDIAGNOSABLE MALADIES.

These cases generally are not recorded because there is so little about them that is tangible. They are perhaps functional derangements simulating organic diseases, sometimes these cases yielding readily to simple remedies, but are puzzling because so evanescent. At other times they are graver, often ending fatally and yielding no facts upon post-mortem examination which will aid in a correct diagnosis. But the list of undiagnosable ailments is rapidly decreasing; for example, Richard Bright in 1827 explained that dropsical effusions are frequently due to diseases of the kidneys; Thomas Addison in 1855 ascribed to disease of the suprarenal capsule the cause of a form of anæmia accompanied by a dingy discoloration of the skin. Until recently zoster frontalis was classified among skin diseases, but now, with much accuracy, it is traceable to disease of Gasser's ganglion. Dr. Tucker related a case in which a lady had been twice badly frightened and her nervous system had been severely prostrated. At intervals she was attacked with an epileptiform seizure, transient paralysis of the entire left half of the body, constriction of the larynx, a state of trance, and finally a trance-like state in which she had died. This patient's mother, after a period of nervous disorder, had become a paraplegic, her father was temporarily insane, the eldest brother has an undefinable nervous disorder and a younger brother spasmodic asthma, while a sister has attacks of recurrent chorea major. In this peculiar group of nervous disorders, what is the underlying cause? It is as yet unknown. This case illustrated the difficulty which hedges about the diagnostication of hundreds of cases of disease.

Dr. Pearson illustrated the difficulties physicians have to overcome in making a diagnosis by reason of the fact that they overlook some points in the history of a case, as in a case in which a patient had swallowed a piece of a metal spoon and it had lodged in the duodenum and blocked up the portal vein.

Dr. W. T. Belfield presented specimens of

ANCHYLOSTOMUM DUODENALE.

These had been referred to him for verification by Dr. R. W. Gelbach, of Mendota, Illinois, who had discovered them in the intestines of young cats that had died of anæmia. Dr. Belfield confirmed Dr. Gelbach's identification of the worms, but for further verification sent them to Dr. Joseph Leidy, of Philadelphia, who replied he thought they were anchylostoma, although he had never seen authentic examples. Dr. Belfield said the anchylostoma are small nematode worms, about half an inch long, which inhabit the duodenums of men and cats, and probably of other animals. Discovered in 1838, their pathogenic significance was recognized by Griesinger in 1851, who found in them the cause of Egyptian chlorosis. They are veritable leeches which fasten themselves to the intestinal mucous membrane and suck the blood of their host. When present in large numbers they induce pernicious and fatal anæmia by exhausting the individual's blood. In tropical climates, particularly Egypt and Brazil, cases of pernicious anæmia or chlorosis produced by them are quite frequent; in Brazil such cases are quickly cured by administering the pulp of fresh figs, which destroys the worms. Quite recently anchylostoma have been searched for and found in patients who died of pernicious anæmia in Germany. So far as Dr. Belfield knew, Dr. Gelbach is the first to discover the worms in the Northern States of the Union. Their presence in cats makes it probable that they infest human beings also in this latitude. The possibility of this cause of pernicious anæmia should therefore be kept in mind, especially when other recognized causes—chronic nephritis, malaria, etc.—cannot be detected.

Dr. E. Andrews asked if these worms were ever found in sufficient numbers to cause death, and he was answered affirmatively.

Dr. W. W. Jaggard exhibited the head of the *tænia mediocannellata* obtained in the practice of Dr. C. G. Smith. The following was the formula used :

| | | |
|---|---|---|
| ℞. | Chloroformii | i.ʒ |
| | Oleores felicis maris | i.ʒ |
| | Ol. figlii | gt. i. |
| | Aquæ camphoræ | ℥ii. |
| | Gum acacia q. s. ft. emuls. | |

